# Quantitative maps of genetic interactions in yeast - Comparative evaluation and integrative analysis

**DOI:** 10.1186/1752-0509-5-45

**Published:** 2011-03-24

**Authors:** Rolf O Lindén, Ville-Pekka Eronen, Tero Aittokallio

**Affiliations:** 1 Biomathematics Research Group, Department of Mathematics, University of Turku, Turku, Finland; 2 Data Mining and Modeling Group, Turku Centre for Biotechnology, University of Turku, Turku, Finland

## Abstract

**Background:**

High-throughput genetic screening approaches have enabled systematic means to study how interactions among gene mutations contribute to quantitative fitness phenotypes, with the aim of providing insights into the functional wiring diagrams of genetic interaction networks on a global scale. However, it is poorly known how well these quantitative interaction measurements agree across the screening approaches, which hinders their integrated use toward improving the coverage and quality of the genetic interaction maps in yeast and other organisms.

**Results:**

Using large-scale data matrices from epistatic miniarray profiling (E-MAP), genetic interaction mapping (GIM), and synthetic genetic array (SGA) approaches, we carried out here a systematic comparative evaluation among these quantitative maps of genetic interactions in yeast. The relatively low association between the original interaction measurements or their customized scores could be improved using a matrix-based modelling framework, which enables the use of single- and double-mutant fitness estimates and measurements, respectively, when scoring genetic interactions. Toward an integrative analysis, we show how the detections from the different screening approaches can be combined to suggest novel positive and negative interactions which are complementary to those obtained using any single screening approach alone. The matrix approximation procedure has been made available to support the design and analysis of the future screening studies.

**Conclusions:**

We have shown here that even if the correlation between the currently available quantitative genetic interaction maps in yeast is relatively low, their comparability can be improved by means of our computational matrix approximation procedure, which will enable integrative analysis and detection of a wider spectrum of genetic interactions using data from the complementary screening approaches.

## Background

The recent advances in experimental biotechnologies have made it possible to start screening genome-wide datasets of quantitative genetic interactions in model organisms such as yeast [1-3]. High-throughput genetic screening approaches, such as those based on epistatic miniarray profiling (E-MAP) [4-7], genetic interaction mapping (GIM) [8], and synthetic genetic array (SGA) [9-11], have provided systematic means to global investigation of quantitative relationship between genotype and phenotype, with potential implications for a wide range of biological phenomena, including, for instance, modularity, essentiality, redundancy, buffering, epistasis, evolution, canalization and development of human disease [1-3,12-21]. The rapid accumulation of quantitative genetic interaction data is providing us with unique opportunities to decipher how genes function as networks to regulate cellular processes and to maintain mutational robustness. However, the massive datasets also call for principled modelling frameworks and efficient analytic approaches to take a full advantage of the in-depth information encoded in the available and emerging quantitative interaction datasets [22]. In particular, efficient bioinformatics procedures enabling integrative analysis of multiple datasets from various screening approaches could increase the quality and coverage of the genetic interaction maps, with the aim of completing the genetic interaction networks in yeast and other organisms.

Comparing the results from the alternative experimental approaches is crucial for validating the observed interactions, estimating the biases related to each approach, and filling the gaps in the currently incomplete datasets. It is therefore likely that comprehensive mapping of the quantitative genetic interaction networks will require integration of a number datasets from different screening approaches, similar to the recent efforts to complete the physical protein-protein interaction (PPI) networks in yeast and human [23-28]. A major challenge in such integrative analysis is that quantitative interaction data generated with the complementary experimental approaches in different laboratories are not directly comparable, due to differences, for instance, in experimental designs, growth conditions or screening protocols as well as in data pre-processing or scoring options. Even when the same mutant pairs are considered, the technical variation can lead to some disagreement in the detection results and to relatively large inconsistency between the datasets in general [8,11]. The correction for such discrepancy can be beyond the capacity of the customized data processing techniques used within the individual screening approaches [29,30]. A common modelling framework, adjusted for the different screening approaches, could improve the comparability of the results and allow for integrative analysis.

Compared to PPI networks, an additional challenge originates from the quantitative nature of the genetic interaction datasets; instead of comparing the overlap in binary terms, such as presence or absence of a physical interaction, here we should take into account the full spectrum of genetic interactions, ranging from extreme cases of negative interactions (i.e., synthetic sick and lethality) to the positive classes of interacting pairs (e.g., masking and suppression subcategories) [2,3,17]. We have recently shown that the quantitative data matrices obtained from the individual quantitative screening approaches can capture different portions of this spectrum, as compared to known classes of genetic interactions; for instance, the SGA and GIM datasets captured relatively well the negative classes of interactions, whereas the prediction of the positive interactions proved much more challenging when using the provided double-mutant fitness data alone [31]. Similar observations have been made also when using the highly processed E-MAP data [32,33]. To improve the predictive power of the individual quantitative datasets, we further developed our computational matrix approximation strategy [34], and showed that it could transform the original fitness matrices so that these allow for better discrimination of not only negative but also the positive end of interaction spectrum from the background variability [31].

In the present study, toward combining the quantitative detections from multiple large-scale genetic interaction approaches, we investigated the consistency among the currently available quantitative interaction datasets in yeast, as well as the sensitivity and specificity of the genetic interactions detected by using the three screening approaches (SGA, GIM and E-MAP), with respect to their overlap in common mutant pairs and coverage of known interacting pairs, as extracted from a gold-standard reference database of genetic interactions (BioGRID). We first show that the comparability of the detections between the different approaches can be improved using standardized matrix-based modelling framework within each individual dataset. Using appropriate scoring and aggregation functions, we then demonstrate how the detections from the different screening approaches can be combined more effectively, compared to that when using the individual datasets alone, suggesting that the matrix approximation-based meta-analytic procedure allows for the full exploitation of the existing data when predicting novel interactions or designing new experiments. To promote its widespread usage in the future screening studies, we have made publicly available an efficient, stand-alone R-implementation of the quantile-based matrix approximation procedure (QMAP), which includes a number of user-adjustable options that can be used to fine-tune the procedure for any given experimental dataset.

## Results and Discussion

### Scoring of quantitative genetic interactions

We have previously introduced a matrix-based modelling and approximation framework, and showed that it provides a quantitative and efficient means for scoring genetic interactions among thousands of genes, thereby leading to improved detection of both positive and negative pairs of interactions in large-scale quantitative screening experiments [31,34]. Briefly, the matrix approximation strategy is based on the observation that most gene pairs in the large-scale genetic interaction screens have no significant interaction with each other [2,3]. This implies that the single-mutant fitness effects, which are needed in the interaction scoring, could be estimated using solely the information encoded in the observed, double-mutant fitness matrix **W**, with entries *w*_*ab *_corresponding to the *m *query and *n *array strains, respectively, that is, *a *= 1,2,...*m *and *b *= 1,2,...*n*. The underlying idea of the matrix approximation it to decompose the original fitness matrix into separate components, **W **= **x **⊗**y**, where the *m *and *n*-dimensional vectors **x **and **y **model the variability across the array and query mutants, respectively [31,34].

In the symmetric case, that is , the above equation expresses in matrix notation the well-established multiplicative null model, *w*_*ab *_= *w*_*a*_*w*_*b*_, which states that the expected neutral phenotype of an organism's fitness, under the null hypothesis that it carries two non-interacting mutations (*a *and *b*), can be estimated by the product of the corresponding single-mutant fitness effects (*w*_*a *_and *w*_*b*_, respectively) [35]. It was shown on symmetric, high-resolution data that the product function is the best null model among a family of alternative models (minimum, additive and log functions), in the sense that it yields a distribution with location close to zero and low dispersion over all of the measured deviations *ε*_*ab *_= *w*_*ab *_- *w*_*a*_*w*_*b *_[35,36]. In the non-symmetric case, *n *≠ *m*, even though the single-mutant effects **x **and **y **are not necessarily equal, these together can provide individual estimates for *w*_*a *_and *w*_*b*_, respectively. In the present work, the estimation of **x **and **y **was performed using a robust, rank-one matrix approximation method, named quantile-based matrix approximation (QMA) [31].

After performing the approximation of the double-mutant fitness matrix **W **under the null multiplicative model, the interaction class of a mutant pair (*a*,*b*) can be predicted using a specific scoring function *s*(*x*, *y*), such as minimum, maximum, product or scaled epistasis [13,35,36], which transform the original fitness matrix into a score (or residual) matrix *s*_*ab *_= *w*_*ab *_- *s*(*x*_*a*_, *y*_*b*_). It has been shown before that there exists effective alternatives to the traditional product function when further classifying the significant genetic interactions into the positive and negative classes [13,31]. Accordingly, the score values *s*_*ab *_can be used in place of the traditional deviations *ε*_*ab *_to test for a genetic interaction between genes *a *and *b*, where a large absolute score provides evidence for genetic interaction, while scores close to zero indicate non-interacting gene pairs. The positive interactions (or alleviating epistatic effects) should result in positive scores (*s*_*ab *_> 0), and the negative interactions (aggravating epistatic effects) in negative scores (*s*_*ab *_< 0), with synthetic lethality being the extreme case (*w*_*ab *_= 0).

Following the lessons learned from the integrative analysis of high-throughput PPI datasets [25], we first evaluated separately the data from the individual screening approaches (SGA, GIM and E-MAP), against a gold-standard reference database of know interactions (BioGRID) [37]. Such within-approach benchmarking resulted in specific parameter combinations for the data-adjusted QMA estimates and scoring functions for positive and negative genetic interaction classes (Additional File 1) [31]. In the following analyses, we utilized these same parameters and scoring functions to assess their robustness, and to demonstrate the relative advantages of the generic matrix approximation strategy, in terms of both improved comparability of the interaction scores as well as integrative detection of genetic interactions, among the screening approaches, in comparison to using the individual datasets alone. Our specific focus here is on the detection of pairs of positive interactions, the accurate scoring of which has been challenging in the past despite the quantitative approaches.

### Agreement between the quantitative datasets

Using the datasets available from three representative screening approaches [5,8,10], we started with pairwise comparisons among the three datasets and characterized the number of common pairs of array and query mutants shared by the datasets (Table 1), as well as the distribution of the known pairs of positive and negative interactions into the data intersections (Table 2). The number of shared mutant pairs was largest in the SGA - E-MAP data pair (184 077 common pairs), second largest in the SGA - GIM data pair (58 215 common pairs), and smallest in the GIM - E-MAP data pair (12 461 common pairs). To investigate the coverage of the known pairs of genetic interactions in the three datasets, we used the existing information on genetically interacting pairs as available in the gold-standard BioGRID database [37]. For the positive class of interactions, we combined the 'Positive Genetic' and 'Phenotypic Suppression' categories, which are composed of alleviating mutant pairs, and for the negative class of interactions, we merged four of the BioGRID's aggravating categories, namely, 'Negative Genetic', 'Synthetic Growth Defect', 'Synthetic Lethality', and 'Phenotypic Enhancement'.

**Table 1:** Parwise intersections between the three datasets used in the study

	SGA	GIM	E-MAP
**SGA**	3885 × 1712	3881 × 15	543 × 339

**GIM**	23.99%	5918 × 41	733 × 17

**E-MAP**	33.34%	5.14%	743 × 743

The values above the diagonal give the number of array and query mutants in common between each pair of datasets, and the values below the diagonal give the percentage of the overlap when compared to the smaller of the two datasets. The diagonal gives the original dataset dimensions (array mutants × query mutants). Only the non-missing mutant pairs shared by the datasets were included in the comparative analyses. The original studies were as follows: SGA [11], GIM [8], E-MAP [5].

**Table 2:** Coverage of the known genetic interactions in the dataset pairs

	SGA		GIM		E-MAP	
**SGA**	810	4723	82	645	3217	16481

**GIM**	0.14%	1.11%	0	85	141	603

**E-MAP**	1.75%	8.95%	1.13%	4.84%	1607	5297

The values above and below the diagonal give the number and percentage, respectively, of known genetic interactions in each of the pairwise intersections as available from BioGRID (version 3.0.64). Left, positive interactions; Right, negative interactions. Only the unambiguous mutant pairs were used here in the comparative evaluations. The BioGRID interactions extracted from these three datasets under the study were pairwise excluded from the positive and negative classes of reference interactions. The diagonal gives the number of interactions excluded from each of the individual datasets. In addition to the E-MAP dataset used in the present analyses, the BioGRID database contains genetic interactions extracted from three other large-scale screening studies performed with the E-MAP approach [4,6,7]. The three dataset intersections contained such interactions as follows (negative/positive interactions): SGA - GIM 37 (45%)/52 (8%), SGA - E-MAP 755 (23%)/1458 (9%), and GIM - E-MAP: 36 (26%)/46 (8%).

Even if the interactions extracted from the three datasets under study were pairwise deleted from the BioGRID's genetic interaction categories (Table 2), there may remain some bias in these categories toward the E-MAP approach due to the large number of interactions identified in the three other large-scale E-MAP studies [4,6,7]. If these had also been excluded from the comparative analyses, the sizes of the reference positive and negative classes would have become much smaller, hence hindering the comparative evaluations. Due to this potential bias, the interaction detection results for the data pairs other than the SGA - GIM should be interpreted with caution. Moreover, it was not initially expected that the matrix approximation could provide any further improvements in the E-MAP data, since this data has already been heavily pre-processed and custom-scored against an expected fitness [29], resulting in a symmetric and close to zero-centered data matrix [38]. Therefore, we focus here on illustrating the benefits of QMA-based integrative analysis using the detection of positive interactions in the SGA - GIM data pair as our principal case study; however, the full set of results are provided in Additional files 2 - 7.

The correlations between the double-mutant fitness matrices were relatively poor among all the three dataset pairs (Table 3); especially striking is the negative correlation between the SGA and GIM fitness matrices (Pearson correlation -0.099 and Spearman correlation -0.021). Beyond the original fitness matrices, we also evaluated - as for the point of comparison for our QMA-based scoring system - their scored versions using the custom-designed scoring systems in the SGA dataset (referred to as 'SGA custom score') [30], and the median estimate for the single-mutant effects with product scoring function in the GIM and E-MAP datasets (referred to as 'GIM/E-MAP median score') [29]. It was found out, however, that the correlation between the interaction scores between the screening approaches remained relatively low even after such scoring of the individual datasets (Table 3). As expected, the comparability of the originally scored E-MAP dataset did not improve by the use of an additional median scoring, especially when the non-parametric Spearman's rank correlation coefficient was being used as a measure of association between the datasets. In contrast, the SGA and GIM datasets benefited to some extent from their individual scorings.

**Table 3:** Pairwise correlations between the three quantitative datasets

		SGA		GIM		E-MAP	
		Fitness	Score	Fitness	Score	Fitness	Score
**SGA**	Fitness	1.000	0.235	-0.099	0.092	0.268	0.490
	Score	0.243	1.000	-0.073	0.095	0.258	0.491

**GIM**	Fitness	-0.021	0.052	1.000	0.954	0.192	0.191
	Score	0.014	0.056	0.824	1.000	0.194	0.196

**E-MAP**	Fitness	0.152	0.245	0.209	0.219	1.000	0.994
	Score	0.144	0.245	0.201	0.221	0.981	1.000

The values above and below the diagonal give the Pearson and Spearman correlation, respectively, as calculated over the non-missing mutant pairs shared by each dataset pair. Fitness, original double-mutant fitness measurements from a screening approach; Score, custom-designed score provided in the SGA dataset or the median estimate for single-mutant effects with product scoring function in the E-MAP and GIM datasets.

**Figure 1:**
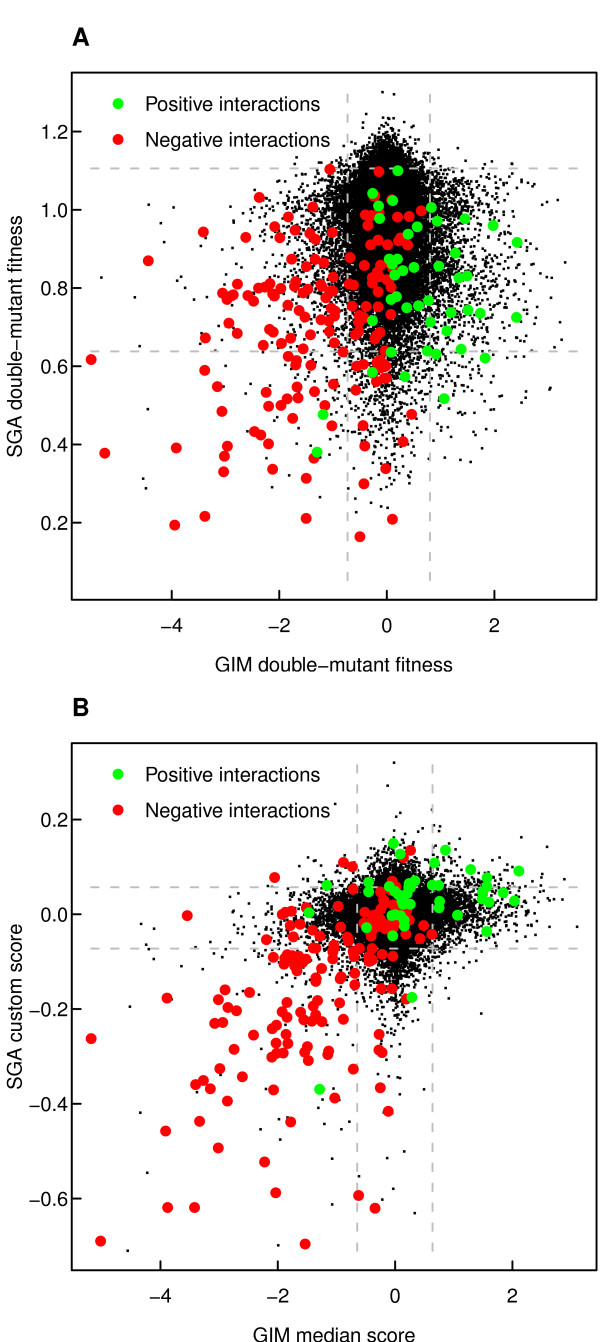
**Scatter plot between the SGA and GIM datasets**. **(A) **Original double-mutant fitness measurements from the two screening approaches. **(B) **Interaction scores: custom-designed score from the SGA data and the median estimate for single-mutant effects with product scoring function in the GIM data. The dotted lines correspond to the extreme 3% level quantiles in the two datasets. The green and red points indicate the positive and negative interactions, respectively, as extracted from the BioGRID database (version 3.0.64, interactions extracted from the SGA and GIM datasets under study were excluded from the two interaction classes).

The reason for the negligible correlation between the SGA and GIM datasets is clearly visible in their scatter plot (Figure 1). It is very difficult to see any apparent patterns of association between the original double-mutant fitness measurements, even for those mutant pairs coding for known genetic interaction (Figure 1A). The custom-scored versions could not provide much improvement in their association, especially for the positive pairs of interactions (Figure 1B). Similar observations were made also in the other dataset pairs (Additional File 2). It should be noted, however, that for reproducible identification of genetic interactions, it suffices that the datasets share similar levels of interaction scores for the most extreme pairs (here the 3% quantiles were used as an expected rate of interactions [11]; Table 2). Similarly, since the ranking of the mutant pairs in terms of their evidence for genetic interactions is of more practical and biological importance, and due to the sensitivity of the Pearson correlation to data transformations and outlier pairs, we will use the more robust Spearman's rank correlation coefficient as our principal measure of association between the quantitative genetic interaction datasets in the next sections.

### Predictive relationship between the datasets

**Figure 2:**
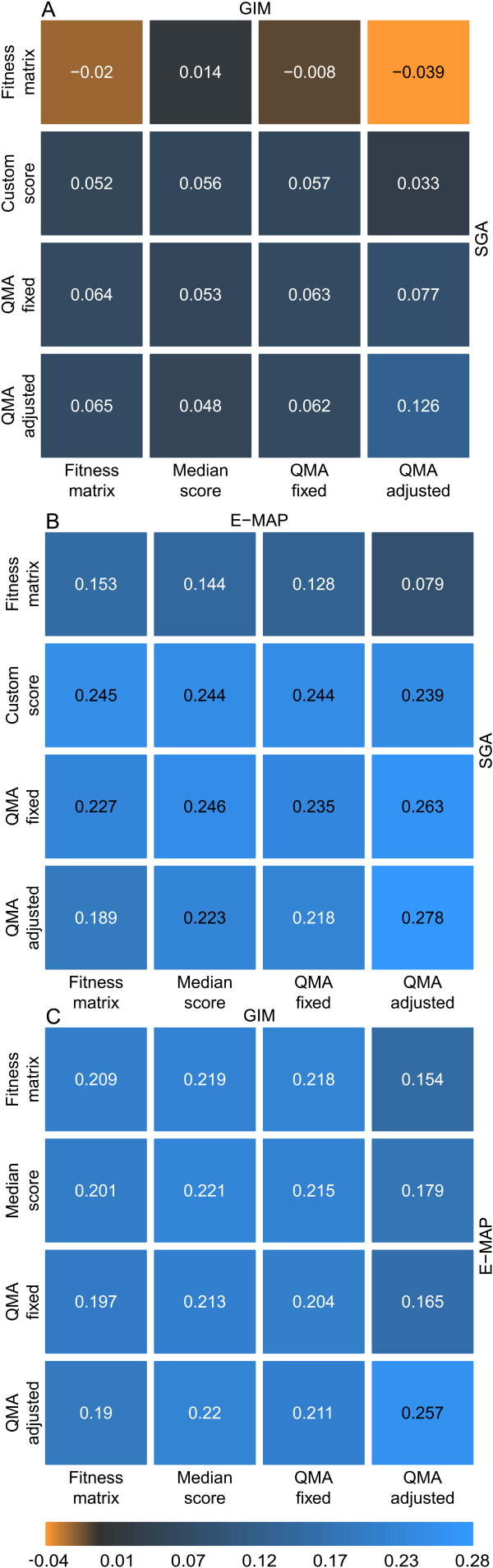
**Pairwise correlations between the three datasets**. **(A) **SGA - GIM data pair, **(B) **SGA - E-MAP data pair, and **(C) **E-MAP - GIM data pair. The Spearman's rank correlation was calculated for different versions of the two datasets: Fitness matrix, original double-mutant fitness measurement; SGA custom score, interaction score provided in the SGA dataset; GIM/E-MAP median score, the median estimate for the single-mutant effects with product scoring function in the GIM/E-MAP data. QMA fixed/adjusted, matrix approximation-based score with its two pre-defined settings for scoring positive interactions (see Additional file 1).

To investigate whether the matrix approximation-based scoring strategy could enhance the between-approach comparability of the quantitative information encoded in the double-mutant fitness matrices, we next used the same estimation parameters and scoring functions defined in the previous within-approach evaluations [31]. Briefly, three parameter combinations for the two QMA parameters were specified per each dataset: one for detecting all the interaction classes simultaneously (referred to as 'fixed setting'), and the others for detecting either the negative or positive classes separately ('adjusted settings'). The scoring functions were also shown to be specific to the alleviating and aggravating interaction classes (Additional File 1). With the use of these QMA-based single-mutant fitness estimation and interaction scoring options, there was an increasing trend in the Spearman's rank correlation coefficient between all the three datasets, when compared to the original double-mutant fitness measurements or the reference scoring approaches, especially when the adjusted QMA setting was used for the positive interactions (Figure 2). Interestingly, with QMA adjusted to negative interactions, the original SGA double-mutant fitness matrix provided better correlation with the GIM dataset than when using the custom-designed SGA scores (Additional File 3).

**Figure 3:**
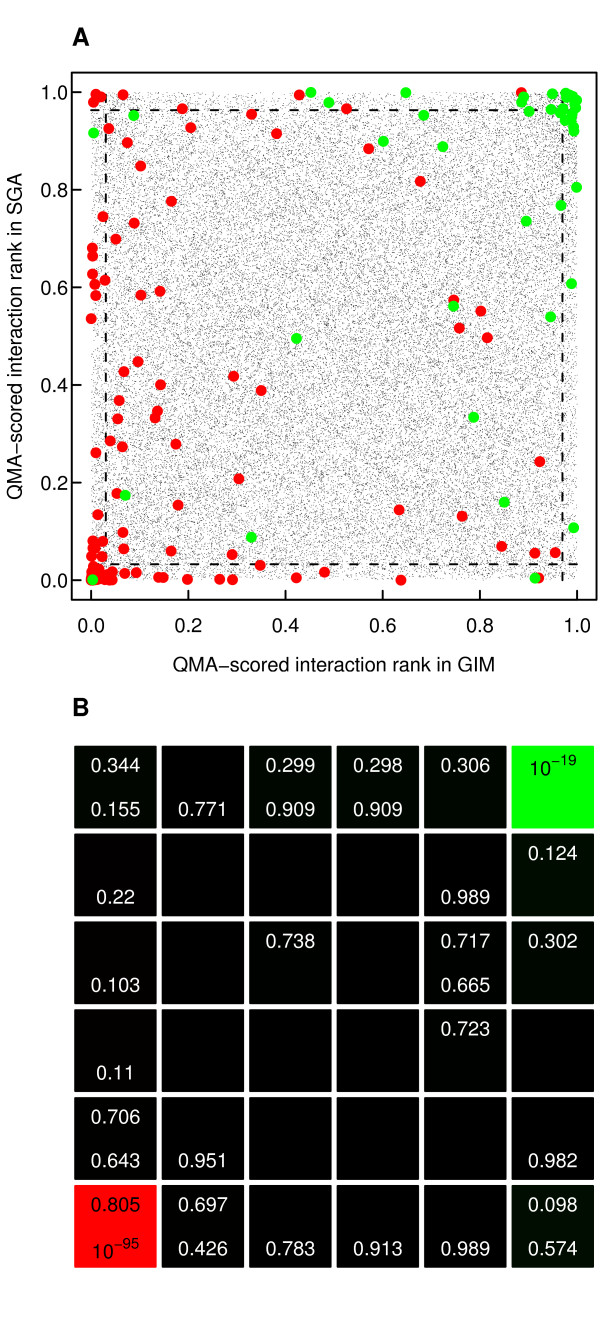
**Rank scatter plot between the SGA and GIM datasets**. **(A) **Interaction score rankings based on the fixed QMA setting for scoring positive interactions. The dotted lines indicate the extreme 3% quantiles in the two datasets. The green and red points indicate the positive and negative interactions, respectively, as extracted from the BioGRID database (version 3.0.64, interactions extracted from the SGA and GIM datasets under study were excluded from the interaction classes). **(B) **Enrichment *p*-values for the positive interactions (upper values, green colouring) and for the negative interactions (lower values, red colouring) in different regions of the scatter plot. Empty cells indicate that the enrichment *p*-value is larger than 0.99.

The relatively low Spearman's rank correlation in the interaction scores between the SGA and GIM datasets is also visible in their rank-based scatter plots (Figure 3A). Even if there were no clear patterns of association in the interactions rankings as a whole, the bulk of the positive pairs were supported consistently by both of the datasets, with only a relatively few interaction pairs near one of the data axes only. Such mutant pairs with discrepancy in their interaction scores may be either due to differences in the screening approaches or due to false positive findings. The interaction pairs lying in the middle of the rank scatter plot are likely to correspond to true non-interaction mutant pairs (Figure 3A). With the negative interaction pairs, there seems to be more variability between the datasets, which may be attributable to the fact that we used here the fixed QMA parameters and scoring functions chosen for positive interactions for illustration purposes. Moreover, the number of known negative interactions is much higher than the number of positive interactions in the datasets (Table 2). Even so, the enrichment of both positive and negative pairs at the extreme corners of the two-dimensional grid was highly statistically significant (Figure 3B). Similar findings were also seen in the other datasets (Additional File 4).

**Figure 4:**
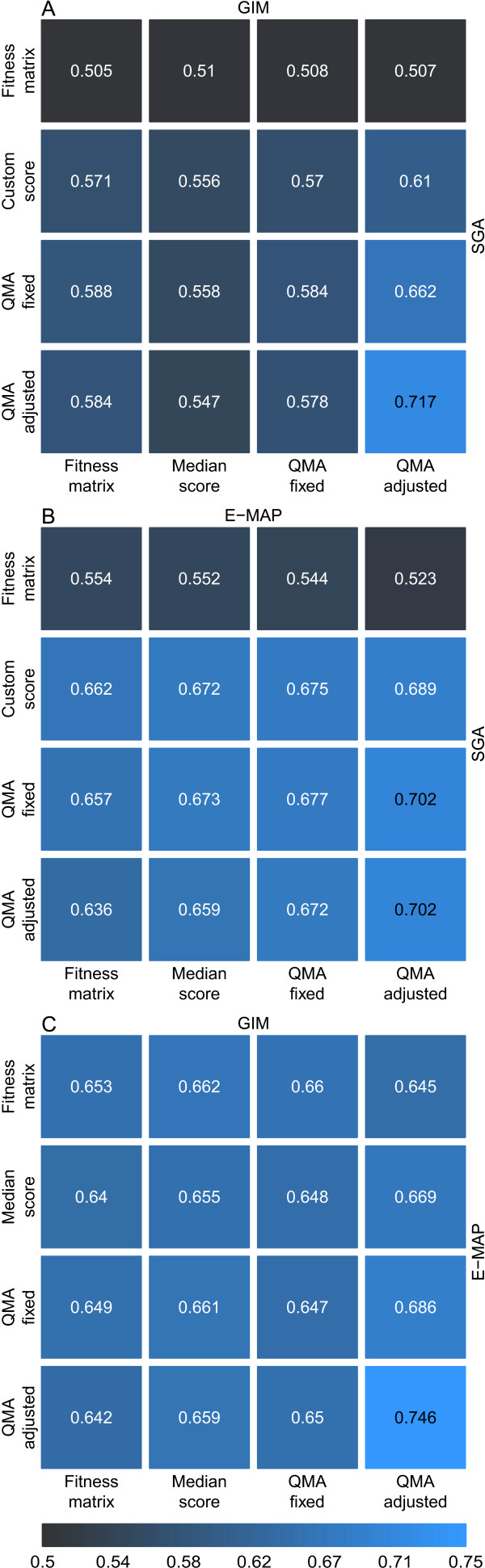
**Pairwise predictive accuracies between the datasets**. The prediction accuracy was assessed using the area under the ROC curve (AUC). when predicting the pairs within 3% of the largest interactions levels in the datasets. **(A) **SGA - GIM data pair, **(B) **SGA - E-MAP data pair, and **(C) **E-MAP - GIM data pair. The AUC value was calculated for different versions of the two datasets: Fitness matrix, original double-mutant fitness measurement; SGA custom score, interaction score provided in the SGA dataset; GIM/E-MAP median score, the median estimate for the single-mutant effects with product scoring function in the GIM/E-MAP data. QMA fixed/adjusted, matrix approximation-based score with its two pre-defined settings for scoring positive interactions (see Additional file 1).

Although the Spearman's correlation is useful for evaluating an overall association between interaction datasets, it may be dominated by the non-interacting pairs near the zero scores, which often are not the most interesting from the biological point of view. To evaluate the agreement between the interaction scores among the most extreme levels, we tested next how accurately one can predict the 3% of the most positive values across the datasets using the same options as in Figure 2. Similarly as with the rank correlation coefficient, the predictive accuracy increased when moving from the original double-mutant fitness values and their custom or median scores to the QMA-based scores using either its fixed or adjusted settings in all the three data pairs (Figure 4). These results demonstrate that it is possible to find such estimation parameters and scoring functions that can markedly improve the prediction of those most extreme positive interaction scores that are shared across the datasets, compared to using the original fitness values or interaction scores only. In the negative classes of interactions, these baseline prediction accuracies were already much higher, especially in those pairs involving the E-MAP dataset, and, accordingly, the benefits of the QMA procedure were not so evident here (Additional File 5).

The modelling framework makes it also possible to avoid performing the single-mutant growth experiments in the large-scale genetic interaction screens, without compromising their quantitative scoring accuracy. Moreover, the model-estimated array-vector was in a good agreement with the experimentally-derived single-mutant fitness measurements available in the SGA data (Spearman's correlation ranged from 0.964 to 0.996, depending whether we use the fixed QMA settings or those adjusted for positive interactions, respectively). Despite such high rank correlation levels, however, there is a significant difference in the location and scaling between the estimated and measured fitness values, indicating that the estimates encode added information for interaction scoring. The QMA settings used here were originally selected on the basis of the pre-release version of the SGA data [31], which contained only 1277 of the query mutations of the current SGA dataset (75%), thus indicating the robustness of the QMA settings. In the following section, we further highlight the potential of the model-based strategy in integrative analysis by using the same QMA setup selected specifically for the positive interactions, even if this will likely to result in compromised prediction accuracies in the negative interaction classes.

### Integrative identification of genetic interactions

After showing that the usage of the matrix approximation-based scoring system in place of the original double-mutant fitness matrix or its custom-scored version can lead to improvements in the comparability between the dataset pairs, we next evaluated whether these observed improvements in the rank correlation or prediction of the extreme pairs could contribute also to improved identification of genetic interactions, when using multiple datasets together, compared to using single datasets alone. To choose an appropriate data integration approach, we first evaluated the predictive performance of four rank aggregation functions (product, minimum, maximum and Borda count, which is effectively the same as the additive function), in terms of how accurately they can detect known pairs of interacting genes. Even if the QMA-based scoring setup was aimed here at the detection of positive interactions, we further tested its prediction capability also for the negative interactions to study its generalization capability beyond the type of interactions it was initially designed for. The prediction performance is illustrated here using the unbiased GIM-SGA data pair, whereas the E-MAP - SGA and E-MAP - GIM pairs are provided in Additional File 6.

**Figure 5:**
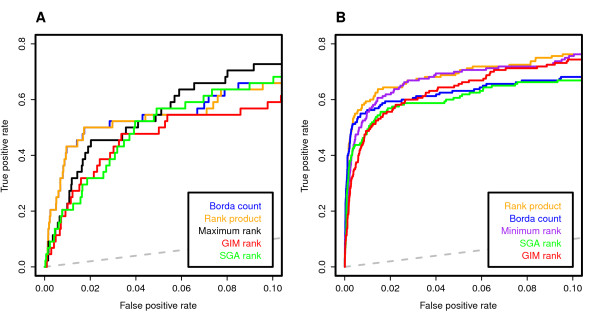
**Integrative prediction of known genetic interactions**. **(A) **positive interactions, **(B) **negative interactions in the SGA - GIM data pair. The true positive rate (TPR or sensitivity) is the fraction of mutant pairs correctly classified into its true interaction class, and the false positive rate (FPR, or 1 - specificity) is the fraction of non-interacting gene pairs incorrectly classified into the particular interaction class. The dotted trace illustrates the average performance of a random classifier. The colour traces indicate the different rank aggregation functions (minimum, maximum, product, and Borda count), which combine the interaction scores across datasets, compared to the ranking of the interaction scores using a single dataset alone (SGA rank, GIM rank, and E-MAP rank). Interaction scores were based on the fixed QMA setting for scoring positive interactions. The minimum function was omitted from the positive interactions and the maximum from the negative interactions for the clarity of illustration. The performance metrics of all the functions are summarized in Table 4.

When combining the interaction scores in the SGA and GIM datasets to detect pairs of positive interactions, the conservative maximum aggregation score gave the best prediction accuracy in terms of the overall AUC (Table 4). However, when focusing on the early sensitivity at the highest specificity levels (or the smallest FPR-levels), which are often more important in many practical applications, the Borda count and rank product were the two best performing methods (Figure 5A). In the detection of the negative interactions in the SGA - GIM dataset pair, the rank product performed better than the Borda count or either of the individual datasets alone (Figure 5B), whereas the liberal minimum rank gave the highest overall AUC performance (Table 4). The good performance of the Borda count and rank product with the positive interactions was also supported by the integrative analysis in the SGA - E-MAP and E-MAP - GIM dataset pairs, especially at the highest specificity levels (Additional File 6). However, the maximum function soon outperformed these two methods when an increasing number of positive interactions were predicted (Table 4, overall AUC). The rank product was found generally best in the prediction of negative interactions in each of the dataset pairs.

**Table 4:** Detection accuracies using the datasets either alone or combined

	Positive genetic interactions			Negative genetic interactions		
Dataset pair	Early sensitivity	Partial AUC	Overall AUC	Early sensitivity	Partial AUC	Overall AUC
**GIM - SGA**						
GIM rank	0.205	0.445	0.794	0.488	0.624	0.872
SGA rank	0.205	0.477	0.785	0.494	0.595	0.795
Borda count	**0.432**	**0.527**	0.826	0.562	0.619	0.857
Minimum rank	0.386	0.496	0.777	0.556	0.663	**0.897**
Maximum rank	0.227	0.525	**0.856**	0.550	0.601	0.804
Rank product	**0.432**	0.522	0.795	**0.594**	**0.680**	0.892

**E-MAP - SGA**						
E-MAP rank	0.345	**0.637**	**0.889**	0.286	**0.510**	0.821
SGA rank	0.148	0.338	0.772	0.208	0.366	0.734
Borda count	**0.348**	0.542	0.868	**0.304**	0.477	0.805
Minimum rank	0.337	0.511	0.832	0.252	0.477	0.825
Maximum rank	0.255	0.575	0.887	0.294	0.460	0.769
Rank product	0.347	0.539	0.854	**0.304**	0.501	**0.826**

**GIM - E-MAP**						
GIM rank	0.300	0.450	0.792	0.237	0.397	0.783
E-MAP rank	0.333	**0.579**	0.884	0.256	0.411	0.767
Borda count	0.367	0.533	0.878	0.293	0.457	0.807
Minimum rank	**0.400**	0.508	0.839	0.298	0.463	0.825
Maximum rank	0.333	0.572	**0.892**	0.279	0.438	0.768
Rank product	0.367	0.530	0.857	**0.326**	**0.480**	**0.827**

Random classifier	0.010	0.050	0.500	0.010	0.050	0.500

The bolded values indicate the best ranking method in terms of the three evaluation metrics: Early sensitivity, true positive rate (sensitivity) at 1% false positive rate (FPR, 99% specificity); Partial AUC, area under the curve (AUC) at 10% FPR; Overall AUC, AUC at 100% FPR. The random classifier accuracies were used as baseline for assessing whether similar accuracies could be obtained by pure chance alone. The rankings provided by the individual datasets (GIM rank, SGA rank and E-MAP rank) were used as reference values for the integrative detection using four different rank aggregation functions (minimum, maximum, product and Borda count). The detection accuracies of the E-MAP ranking alone may be highly overestimated due to a large number of such genetic interaction pairs in the BioGRID that were extracted from the other large-scale E-MAP screening studies (see Table 2).

Taken together the integrative prediction results in the three dataset pairs, the Borda count and the rank product performed equally well when the aim is to identify the first candidate set of positive interactions with the highest specificity for follow-up studies, whereas the more stringent maximum function provided the best prediction accuracy when larger numbers of positive interactions are being identified. In the detection of negative interactions, the intermediate rank product showed consistently the best results among all the data pairs, making it an appropriate rank aggregation function in case both positive and negative interactions are being detected using the same setup. In addition to showing the benefits of the integrative detection, these results can also be used for comparative evaluation of the detection power among the individual datasets from the different screening approaches. For instance, on the basis of the same reference set of known interactions on a common set of shared mutant pairs in the SGA and GIM datasets, the GIM approach seems to detect particularly well larger number of negative interactions (Table 4), whereas the nearly genome-wide SGA dataset provides comparable detection power in the positive end of the genetic interaction spectrum (Figure 5).

Although the integrative detection based on combined scores was shown to provide marked improvements in the detection of both positive and negative interaction classes when using the SGA and GIM datasets together, it was interesting to note that in the SGA - E-MAP dataset pair, the E-MAP data alone provided extremely good detection accuracies in the positive class of interactions (Table 4). Rather than being a result of the superiority of this particular dataset, this is more likely attributable to the fact that many of the pairs (23%) of positive interactions in the BioGRID originate from the other large-scale genetic interaction screens performed with the E-MAP approach [4,6,7] (Table 2). These pairs clearly dominate the joint distribution of the positive interactions, while being supported by the SGA approach to a varying degree (Additional File 4). Interestingly, the detection of the negative interactions by the E-MAP approach alone was found sub-optimal (Table 4). Moreover, the additional benefits gained by the integrative analysis were more pronounced in the GIM - E-MAP than in the SGA - E-MAP data pair (Table 4). These results demonstrate that the intrinsic differences between the screening approaches influence how much they can complement each other.

## Conclusions

To our knowledge, the present study is the first systematic and objective comparative evaluation of data from the main large-scale quantitative genetic interaction screening approaches (SGA, GIM and E-MAP). We showed here that even if the association between the original fitness measurements or their interaction scores is relatively low, their comparability can be improved by means of our matrix approximation technique. Toward an integrative analysis, we showed that a multi-approach analysis of quantitative genetic interactions can provide novel findings which are complementary to those obtained using any single screening approach alone. An integrative analysis can therefore provide a systematic means to pool information from previous interaction studies, with the aim of maximizing the number of both positive and negative interactions without compromising the reliability of the detections, as well as of minimizing the number of additional experiments needed when prioritizing of future screens. In general, such computational approach can facilitate the experimental efforts by improving the quality and coverage of the current genetic interaction networks, towards completing the still incomplete information of genetic interactions in yeast, which is - by and large - complementary to that obtained from the physical protein interactions and complexes [1,5,11,17,39,40].

Although these results already demonstrate the potential of integrating datasets across different screening approaches using the matrix approximation strategy, more comprehensive studies are warranted in the future that combine experimental data from various types of genetic interaction studies, such as those performed under different environmental conditions, using fitness phenotypes other than growth, or on multiple perturbations or study organisms to investigate questions related, for instance, to plasticity and evolution of genetic networks or higher-order and interspecies interactions [2,3,17,41-47]. Although we illustrated here the feasibility of the integrative analysis through QMA with its previously fixed parameters and scoring functions selected for each screening approach individually, even better prediction accuracies will likely to be obtained after a systematic optimization of these options for each dataset combination, downstream analysis objective, and interaction strength level separately (Additional File 7). The efficient QMA R-package, which includes a number of user-adjustable parameters (Additional File 8), was made available here to enable such tailored matrix approximation that meets the needs of a given study.

A potential limitation of the current evaluation setup is the definition of the reference set of interactions using the BioGRID database. For instance, since the interactions in the BioGRID database originate from multiple genetic interaction screening studies, there can be cases where a mutant pair AB is reported as encoding an interaction, even if BA is not, or where the reciprocal pairs AB and BA are marked as belonging to different classes of interactions. To make sure that such cases do not interfere with the comparative evaluations, we filtered out any unambiguous interaction pairs, and for the remaining interactions, we used the same interaction class for the reciprocal mutant pairs. Moreover, to provide as fair assessment as possible, we excluded those interactions identified from the datasets under comparison. Therefore, the detection accuracies presented here should be considered as lower bounds for the true accuracy of the screening approaches or their combination. Even if there may still remain some biases, especially toward the well-represented E-MAP approach, the BioGRID database includes also a wide range of other large-scale studies, thus providing a comprehensive reference set for the evaluations. To improve the future benchmarking studies, it would be beneficial to add a specific category for known non-interacting mutant pairs, similar to that available for physically non-interacting protein pairs [48].

Analogous to efforts for completing the mapping of the physical PPI networks [23-28], it would be important to provide the community with an easy access also to the raw interaction datasets, similar to that provided in the SGA database DRYGIN [30]. For instance, our matrix approximation procedure was much more efficient with the original double-mutant fitness measurements, as provided by the SGA and GIM laboratories, compared to the highly processed and scored E-MAP datasets. The results with the E-MAP being one of the datasets were in many cases drastically different from that with the SGA - GIM dataset pair. As with any high-throughput assays, the large-scale genetic screening approaches are inherently noisy and biased in their nature, suggesting that each single assay can reveal only a limited scope of the full spectrum of genetic interaction classes. Therefore, it is likely that integrative analysis of data from the complementary screening approaches will be essential to complete the quantitative genetic interaction networks in yeast and other organisms. We invite those participating in the genetic interaction mapping effort to try out the matrix approximation-based procedure and to give us input and suggestions for its further improvements.

## Methods

The methodological aim of the present study was to enable an integrated analysis of multiple genetic interaction datasets using a common scoring framework. adjusted for the high-throughput quantitative screening approaches. The next sections describe the genetic interaction datasets used to demonstrate the benefits of such integrative approach, as well as the methods used to model, standardize, compare and merge these datasets, while maintaining their biological consistency and quantitative nature.

### Genetic interaction matrices

Three large-scale quantitative data sets on yeast were used in the present work for the systematic and comparative evaluations. To investigate the potential limitations in the between-approach agreement and relative benefits gained by an integrative analysis among the currently available high-throughput quantitative genetic interaction maps, we chose representative example datasets across the spectrum of high-throughput interaction screening approaches currently used for *Saccharomyces cerevisiae*.

#### E-MAP dataset

The first dataset was available from the epistatic miniarray profiling (E-MAP) study of quantitative genetic interactions between genes involved in yeast chromosome biology [5]. The original fitness measurements among 754 alleles of 743 genes were highly filtered and processed, providing a symmetric data matrix with close to zero-centered quantitative distribution for the pairwise interaction scores [29,49]. The raw, unprocessed double-mutant fitness measurements were not available from this study.

#### GIM dataset

Representing another screening approach, the genetic interaction mapping (GIM) combines ideas from the synthetic lethality analysis by microarray (SLAM) [50,51] and from synthetic genetic array (SGA) approaches [9,10]. The data matrix available from its pilot study contains double-mutant fitness measurements among 5918 array and 73 query genes [8]. The filtered fitness effects were transformed back to non-log-scale to produce quantitative distribution with mean and median close to unity.

#### SGA dataset

The third and the largest of the datasets is available from the recent SGA screening study [11]. This data set contains double-mutant fitness measurements among 3885 array and 1712 query genes. The filtered and normalized double-mutant fitness data matrix, with median close to unity, was used in the matrix approximation procedure. The same dataset also includes a customized SGA scoring of the gene pairs [30,52], which was used here as a baseline value for our QMA-based scoring procedure.

#### Matrix approximation

The quantile-based matrix approximation (QMA) is an efficient rank-one matrix approximation method, which is conceptually similar to the Tukey's median polish procedure, except that QMA uses multiplicative model instead of additive model and quantiles instead of medians [31]. More specifically, the estimation of the single-mutant fitness effects is based on sub-sequent calculation of the *p *and *q*-quantile points for the rows and columns of the double-mutant fitness matrix **W**, respectively, and then arranging these quantiles in the estimated array and query vectors **x **and **y**.

#### Scoring of interactions

The presence and sign of an epistasis interaction between a gene pair (*a*,*b*) was scored using the residual *s*_*ab *_= *w*_*ab *_- *s*(*x_a_*, *y*_*b*_). To avoid potential bias among the different genes in the datasets, duplicate rows and columns in the double-mutant fitness matrices were combined by calculating mean over the duplicates. The final dimensions of the data matrices are shown in Table 1. Before the data integration, each of the double-mutant fitness matrices was scored separately using the default QMA settings and scoring functions (Additional File 1), as described before [31].

#### Ranking of interactions

A gene pairs (*a*,*b*) was ranked according to its interaction score *s*_*ab *_obtained in each individual dataset using the fixed QMA settings and scoring functions for positive interactions (Additional File 1). A rank-based data aggregation was used for robust integration of the scores from two screening approaches. More precisely, four rank aggregation functions (minimum of the ranks, maximum of the ranks, product of the ranks, and Borda count, which is effectively the sum of the ranks) were evaluated in terms of their accuracy, compared to using the rankings from a single dataset alone.

### Evaluation setup and measures

The pairwise intersections between the three dataset pairs were evaluated separately in terms of their number of common array and query mutants (Table 1), the coverage of the known pairs of genetic interactions (Table 2), as well as their association in fitness values and interaction scores across the shared mutant pairs (Table 3). The shared intersection among all the three datasets was only 498 × 7 in size, including 178 known negative and only 31 known positive interactions from the BioGRID database. Therefore, this triple intersection could not be reliably evaluated here.

#### BioGRID interaction matrix

We used the interactions available in the gold-standard BioGRID database (version 3.0.64 for *S. cerevisia*e) [37]. We constructed a BioGRID's interaction matrix by treating the gene pairs extracted from the database as unordered, meaning that if an interaction exists for a mutant pair AB, we also copied the same interaction for the mutant pair BA for biological consistency. Similar symmetric strategy has been used also in previous studies [4-7,11,31]. For each pairwise intersection between datasets, separate positive and negative interaction matrices were created for evaluation purposes.

#### BioGRID interaction classes

Positive interaction matrix is constructed using 'Phenotypic Suppression' and 'Positive Genetic' categories from BioGRID database, and negative interaction matrix was generated by combining 'Synthetic Lethal', 'Synthetic Growth Defect', 'Phenotypic Enhancement' and 'Negative Genetic' categories. Such interaction matrices are ternary matrices with entries representing either an interacting, non-interacting or ambiguous case, where the pair belongs to both interaction classes. Since the ambiguous cases can lead to biases in the evaluation results, they were excluded from the evaluations.

#### Agreement between the datasets

The congruence between the dataset pairs was evaluated by calculating the Pearson and Spearman correlations across those mutant pairs shared by both datasets. The agreement of the datasets in terms of their extreme fitness values or interaction scores was evaluated by constructing interaction matrices using one of the datasets to define positive and negative genetic interactions. We used extreme 3% of the mutant pairs, according to the interaction rate estimate based on unbiased screens (3.15% [11]), and the BioGRID interactions here among the three dataset intersections (2.99%; Table 2). Other cut-off levels (1% and 5%) were also considered (Additional File 7).

#### Receiver operating characteristics

The receiver operating characteristic (ROC) curves were used to assess the discovery rate of genetic interactions. A single ROC curve summarizes the trade-off between true positive rate (TPR) and false positive rate (FPR) on a ranked list of mutant pairs. The true and false interactions were defined here using the interaction matrices (from the BioGRID or using 3% extreme values). The overall prediction performance was summarized using the area under the ROC curve (AUC). For an ideal classifier, TPR = 1, FPR = 0 and AUC = 1, whereas a random classifier has on average AUC of 0.5.

#### Partial AUC and early sensitivity

In many practical application cases, only the first few candidate mutant pair can be followed-up in further validation studies. Therefore, it is important to evaluate also the performance of a mutant pair ranking at low FPR levels, that is, for those pairs with highest specificity. We used here the partial area under the ROC curve (pAUC), in which the range of FPR is limited to a predefined interval between zero and *r *(here *r *= 0.1), and the resulting area is then normalized by dividing it with *r*. To investigate the early sensitivity of the detections, we also calculated the TPR at FPR of 0.01.

#### Enrichment of genetic interactions

To test the enrichment of interactions over random in different parts of scatter plots, the plots were divided into six-by-six grid. For each of these 36 parts, we performed a standard hypergeometric test to calculate the enrichment *p*-values for positive and negative interactions separately:

Here, *K *is the total number of gene pairs in the grid, *M *is the total number of (positive or negative) interactions (*M *≤ *K*), *m *is the number of interactions found (*m *≤ *M*), and *t *is the number of gene pairs in the particular grid cell (*m *≤ *t *≤ *K*). The *p*-values in the figures were limited between 10^-100 ^and 0.99.

### Implementation issues

To promote its widespread usage in the future screening studies, we have made publicly available an efficient, stand-alone R-implementation of the quantile-based matrix approximation procedure (QMAP). This implementation includes a number of user-adjustable options that can be adjusted through a graphical user interface to fine tune the procedure for a given experimental dataset and downstream analysis object under investigation. Along with the open source R-code, the implementation contains documentation of the data format for the input data, the parameters of the various options, as well the output data of the QMAP (Additional File 8).

## List of abbreviations

AUC: area under the curve; E-MAP: epistatic miniarray profiling; FPR: false positive rate; GIM: genetic interaction mapping; PPI: protein-protein interaction; partial area under the curve (pAUC); QMA: quantile-based matrix approximation; SLAM: synthetic lethality analysis by microarray; ROC: receiver operating characteristic; SGA: synthetic genetic array; TPR: true positive rate.

## Authors' contributions

TA conceived the study, and ROL participated in its design. ROL and VPE developed and implemented the matrix approximation method. ROL analyzed the datasets. ROL and TA wrote the manuscript. All authors read and approved the final manuscript.

## Supplementary Material

Additional file 1 **The pre-defined QMA settings used with the different screening approaches and interaction classes**. Click here for file

Additional file 2 **Scatter plots of the fitness values and interaction scores in the SGA - E-MAP and E-MAP - GIM data pairs**. Click here for file

Additional file 3 **Pairwise correlations between the three datasets when using scoring functions for negative interactions**. Click here for file

Additional file 4 **Rank-based scatter plots and enrichment *p*-values for the SGA - E-MAP and E-MAP - GIM data pairs**. Click here for file

Additional file 5 **Pairwise predictive accuracies between the datasets with scoring functions for negative interactions**. Click here for file

Additional file 6 **Integrative prediction of known genetic interaction classes in the SGA - E-MAP and E-MAP - GIM data pairs**. Click here for file

Additional file 7 **Pairwise predictive accuracies between the datasets when using 1% and 5% cut-off thresholds for interactions**. Click here for file

Additional file 8 **An efficient, stand-alone R-implementation of the quantile-based matrix approximation procedure (QMAP)**. Click here for file
