# Supplementary material for: Quantitative maps of genetic interactions in yeast - Comparative evaluation and integrative analysis
Source: BMC Syst Biol. 2011 Mar 24;5:45. doi: 10.1186/1752-0509-5-45 (PMC3079637; doi:10.1186/1752-0509-5-45)
Supplement: Additional file 1 — The pre-defined QMA settings used with the different screening approaches and interaction classes. [file 1752-0509-5-45-S1.PDF]

The QMA settings used with the different screening approaches and interaction classes.

Screening approach/ QMA setting	QMA parameters (p,q)	Pre-processing option	Positive interactions	Negative interactions
SGA				
Fixed setting	(0.55,0.95)	No	Product	Minimum
Adjusted Positive	(0.10,0.95)	No	Product	
Adjusted Negative	(0.95,0.50)	No		Minimum
GIM				
Fixed setting	(0.60,0.50)	No	Minimum	Scaled epistasis
Adjusted Positive	(0.05,0.95)	No	Maximum	
Adjusted Negative	(0.80,0.25)	No		Scaled epistasis
E-MAP				
Fixed setting	(0.50,0.60)	Row mean	Minimum	Minimum
Adjusted Positive	(0.30,0.65)	Row mean	Minimum	
Adjusted Negative	(0.50,0.15)	Row mean		Minimum

The QMA parameters, pre-processing options, and scoring functions were optimized for the three screening approaches and for the two interaction classes, resulting in three combinations per dataset: one for scoring both positive and negative interactions (Fixed setting), and the others for scoring the positive and negative interactions separately (Adjusted Positive and Negative, respectively). These scoring setups are from the previous investigation (Eronen VP, Lindén RO, Lindroos A, Kanerva M, Aittokallio T: Genome-wide scoring of positive and negative epistasis through decomposition of quantitative genetic interaction fitness matrices. PLoS One 2010, 5:e11611). In the present study, the same pre-processing options were used. For illustration purposes, the QMA parameters obtained with the fixed setting and the scoring functions for positive interactions were mainly used in the present study.
