# Supplementary material for: Quantitative maps of genetic interactions in yeast - Comparative evaluation and integrative analysis
Source: BMC Syst Biol. 2011 Mar 24;5:45. doi: 10.1186/1752-0509-5-45 (PMC3079637; doi:10.1186/1752-0509-5-45)
Supplement: Additional file 2 — Scatter plots of the fitness values and interaction scores in the SGA - E-MAP and E-MAP - GIM data pairs. [file 1752-0509-5-45-S2.PDF]

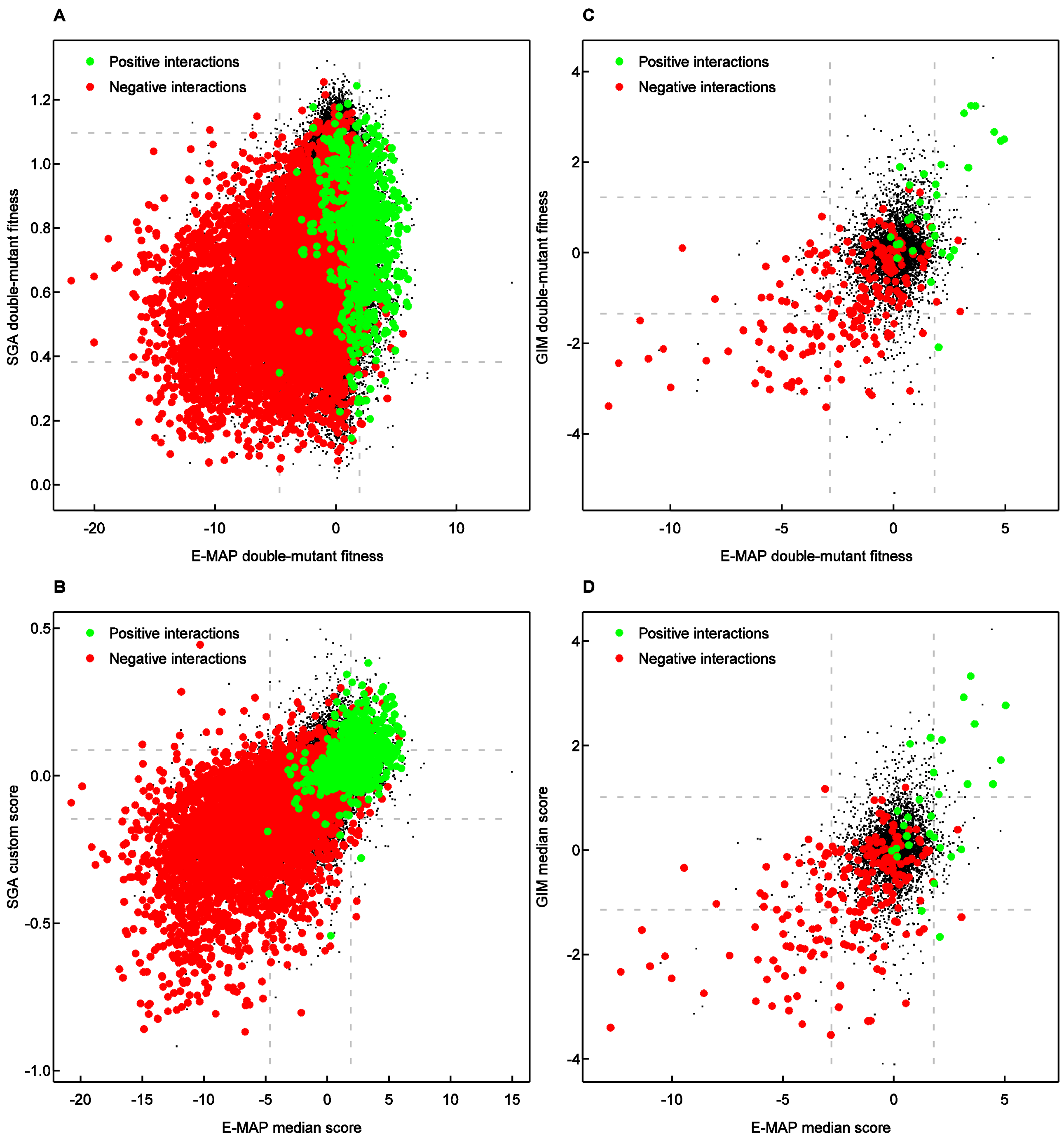

Scatter plots between the fitness and scores in SGA – E-MAP and E-MAP – GIM data pairs.

Left: **(A)** Original double-mutant fitness measurements, and **(B)** custom-built score in the SGA data and the median estimate for single-mutant effects with product scoring function in the E-MAP data.

Right: **(A)** Original double-mutant fitness measurements, and **(B)** the median estimate for single-mutant effects with product scoring function both in the E-MAP and GIM datasets (see Table 3). The dotted lines correspond to the extreme 3% level quantiles in the two datasets. The green and red points indicate the positive and negative interactions, respectively, as extracted from the BioGRID database (version 3.0.64, interactions extracted from the datasets under study were excluded here).
