# Supplementary material for: Quantitative maps of genetic interactions in yeast - Comparative evaluation and integrative analysis
Source: BMC Syst Biol. 2011 Mar 24;5:45. doi: 10.1186/1752-0509-5-45 (PMC3079637; doi:10.1186/1752-0509-5-45)
Supplement: Additional file 3 — Pairwise correlations between the three datasets when using scoring functions for negative interactions. [file 1752-0509-5-45-S3.PDF]

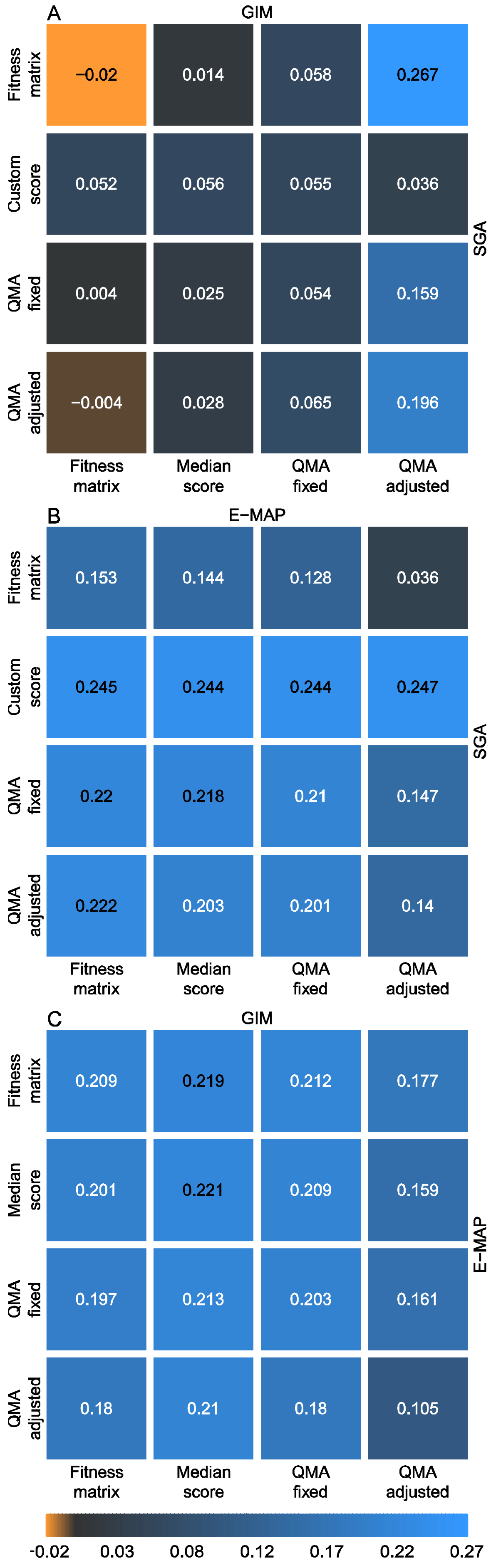

Pairwise correlations between the datasets with scoring functions for negative interactions.

(A) SGA-GIM data pair, **(B)** SGA-E-MAP data pair, and **(C)** E-MAP-GIM data pair. Spearman’s rank correlation was calculated for different versions of the two datasets: Fitness matrix, original double-mutant fitness measurement; SGA custom score, interaction score provided in the SGA dataset; GIM / E-MAP median score, the median estimate for the single-mutant effects with product scoring function in the GIM/E-MAP data. QMA fixed/adjusted, matrix approximation-based score when using the two pre-defined settings for scoring negative interactions (see Additional file 1).
