# Supplementary material for: Quantitative maps of genetic interactions in yeast - Comparative evaluation and integrative analysis
Source: BMC Syst Biol. 2011 Mar 24;5:45. doi: 10.1186/1752-0509-5-45 (PMC3079637; doi:10.1186/1752-0509-5-45)
Supplement: Additional file 4 — Rank-based scatter plots and enrichment p-values for the SGA - E-MAP and E-MAP - GIM data pairs. [file 1752-0509-5-45-S4.PDF]

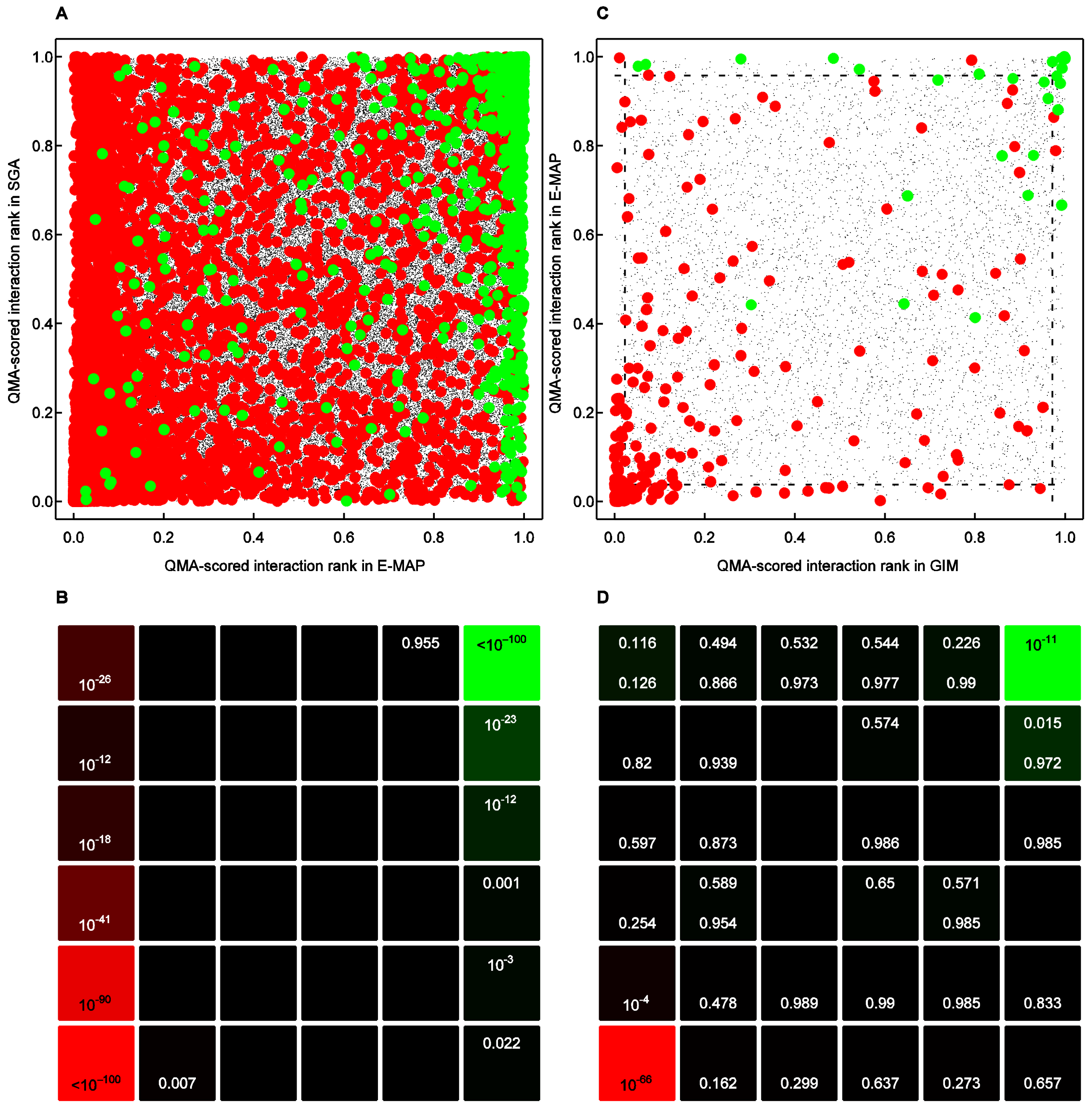

Rank scatter plots and enrichment *p*-values for the SGA – E-MAP and E-MAP – GIM pairs. *Upper row:* Interaction score rankings in **(A)** SGA-E-MAP data pair and **(C)** E-MAP – GIM pair. The scoring was based on the fixed QMA setting for scoring positive interactions. The dotted lines indicate the extreme 3% quantiles in the two datasets. The green and red points indicate the positive and negative interactions, respectively, as extracted from the BioGRID database (version 3.0.64, interactions extracted from the datasets under study were excluded from the interaction classes). *Bottom row:* Enrichment *p*-values for the positive interactions (upper values, green colouring) and for the negative interactions (lower values, red colouring) in the **(A)** SGA-E-MAP dataset pair and **(C)** E-MAP – GIM pair. The empty cells indicate that the enrichment *p*-value is larger than 0.99.
