# Supplementary material for: Quantitative maps of genetic interactions in yeast - Comparative evaluation and integrative analysis
Source: BMC Syst Biol. 2011 Mar 24;5:45. doi: 10.1186/1752-0509-5-45 (PMC3079637; doi:10.1186/1752-0509-5-45)
Supplement: Additional file 5 — Pairwise predictive accuracies between the datasets with scoring functions for negative interactions. [file 1752-0509-5-45-S5.PDF]

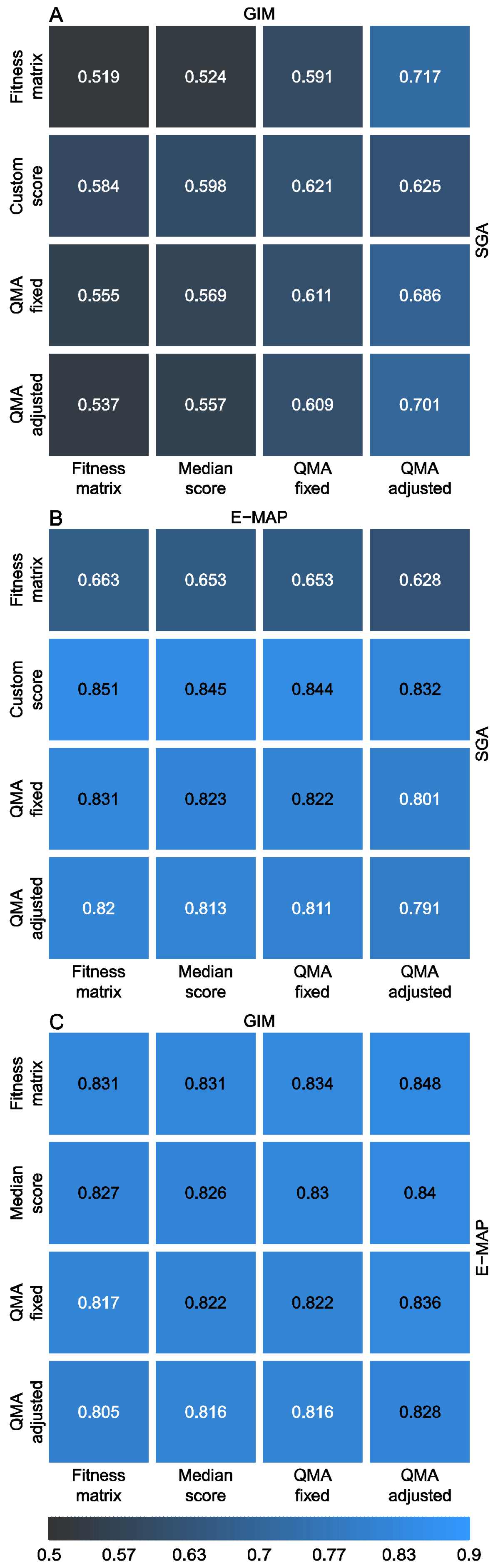

Predictive accuracies between the datasets with scoring functions for negative interactions.

(A) SGA-GIM dataset pair, **(B)** SGA-E-MAP dataset pair, and **(C)** E-MAP-GIM dataset pair. Prediction accuracies were assessed using the area under the ROC curve (AUC) when predicting the pairs within 3% of the largest interactions levels between the datasets. The AUC value was calculated for different versions of the two datasets: Fitness matrix, original double-mutant fitness measurement; SGA custom score, interaction score provided in the SGA dataset; GIM/E-MAP median score, the median estimate for the single-mutant effects with product scoring function in the GIM/E-MAP data. QMA fixed/adjusted, matrix approximation-based scoring system with the two pre-defined settings for scoring negative interactions (see Additional file 1).
