# Supplementary material for: Quantitative maps of genetic interactions in yeast - Comparative evaluation and integrative analysis
Source: BMC Syst Biol. 2011 Mar 24;5:45. doi: 10.1186/1752-0509-5-45 (PMC3079637; doi:10.1186/1752-0509-5-45)
Supplement: Additional file 6 — Integrative prediction of known genetic interaction classes in the SGA - E-MAP and E-MAP - GIM data pairs. [file 1752-0509-5-45-S6.PDF]

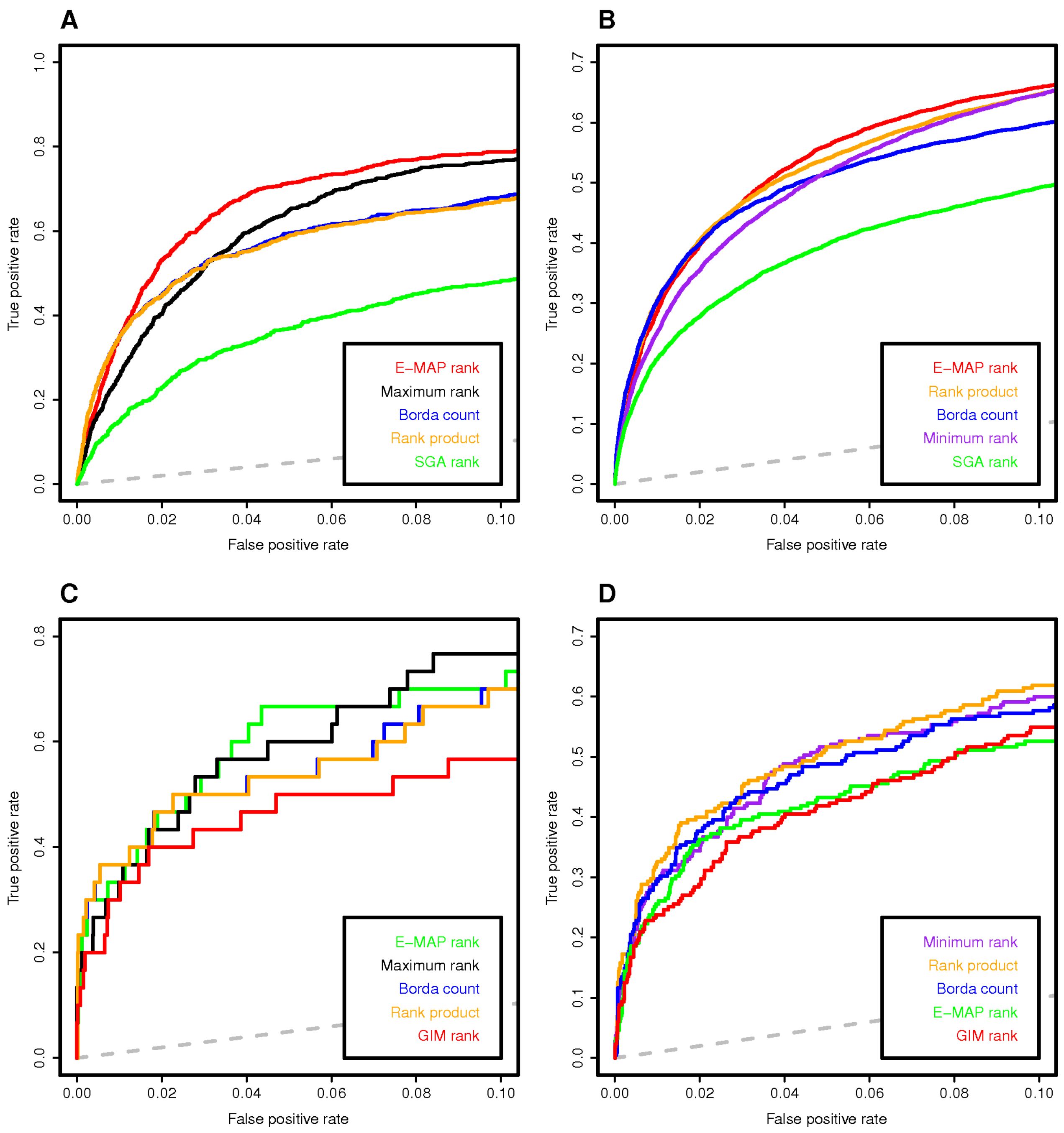

Integrative prediction of known genetic interactions in the E-MAP – SGA and E-MAP – GIM dataset pairs.

Left: Positive interactions for **(A)** E-MAP – SGA dataset pair, and **(C)** E-MAP – GIM pair.

Right: Negative interactions for **(B)** E-MAP – SGA dataset pair, and **(D)** E-MAP – GIM pair. The true positive rate (TPR or sensitivity) is the fraction of mutant pairs correctly classified into its true interaction class, and the false positive rate (FPR, or 1 - specificity) is the fraction of non-interacting gene pairs incorrectly classified into the interaction class. The dotted trace illustrates the average performance of a random classifier. The colours indicate the different rank aggregation functions (minimum, maximum, product, and Borda count), which all combine the interaction scores across datasets, compared to the ranking of the interaction scores within single dataset alone (GIM rank, E-MAP rank and SGA rank). Interaction scores were based on the fixed QMA setting for scoring positive interactions. The minimum function was omitted from the positive interactions and the maximum from the negative interactions for clarity of illustration. The performance metrics of all the functions are summarized in Table 4.
