# Supplementary material for: Quantitative maps of genetic interactions in yeast - Comparative evaluation and integrative analysis
Source: BMC Syst Biol. 2011 Mar 24;5:45. doi: 10.1186/1752-0509-5-45 (PMC3079637; doi:10.1186/1752-0509-5-45)
Supplement: Additional file 8 — An efficient, stand-alone R-implementation of the quantile-based matrix approximation procedure (QMAP). [file 1752-0509-5-45-S8.ZIP › read me.pdf]

Quantile matrix approximation (QMA)

Description

Provides an efficient, robust implementation to calculate rank-one matrix approximations for quantitative genetic fitness matrices using quantiles. Using this package, one can also generate preprocessed and scored matrices to aid detection of both positive and negative genetic interactions.

Installation

On command prompt / console type:

```
R CMD INSTALL "/path/to/package/QMA_1.0.tar.gz"
```

Usage

```
getDataset(datasetCase, scoringFunction, data.ind, processing, params,
fitnessMatrix)

QMEstimate(fitness, p, type)
```

Arguments

datasetCase

Set of different presets for params variable. Presets here bypass any values given to 'params' .

1 = original matrix defined by parameter 'fitnessMatrix',

2 = residual matrix for median estimate,

3 = QMA estimate with p1 and p2 defined by 'params',

4 = SGA QMA fixed for positive genetic interactions (PGI),

5 = SGA QMA adjusted for PGI,

6 = GIM QMA fixed for PGI,

7 = GIM QMA adjusted for PGI,

8 = E-MAP QMA fixed for PGI,

9 = E-MAP QMA adjusted for PGI.

scoringFunction

Scoring used for the matrix.

1 = Traditional product scoring (default),

2 = minimum scoring,

3 = maximum scoring,

4 = scaled epistasis.

data.ind

Way of combining array and query estimates with the fitness matrix.

"aq" = use both array and query estimates (default),

"aa" = use array estimate for both dimensions,

"qq" = use query estimates for both dimensions.

processing

Defines the used preprocessing option.

0 = Substract f(fitnessMatrix) from fitnessMatrix,

1 = Substract f(rows) from fitnessMatrix,

2 = Substract f(columns) from fitnessMatrix,

3 = Return the original matrix,

4 = scale each column st. f(row(fitnessMatrix)) maps to one and f.lb(row(fitnessMatrix)) maps to zero,

5 = same as 4 but for columns.

params

Parameters for calculation of QMA estimate. Should be a list containing variables p1 and p2 with values between 0 and 1. To use this parameter, set datasetCase to value 3. Example: params = list(p1 = 0.123, p2 = 0.456).

fitnessMatrix

Matrix that is used as the base of calculations. Variable can be either a file name or a R object. If fitnessMatrix is a file name, the file must either be a CSV or RDATA file. In the case of an RDATA file, the file must contain a variable called 'fitnessMatrix'. The matrix must have unique row and column labels in order to be handled properly.

fitness

Fitness matrix for which QMA estimate is generated.

p

Quantile positions for array and query estimates in QMA method. Two element numeric vector with values between zero and one.

Value

Output data is a list, that contains the preprocessed, estimated and scored matrix 'm', single-mutant estimates 'array' (for rows) and 'query' (for columns), 'parameters' with parameters passed to the function, and 'fitness.name' containing an identifying name for the generated data set.

Authors

Ville-Pekka Eronen, Rolf Lindén

Examples

```
# Example 1: Generate a rank-one estimate
# and residual matrix from volcano dataset.
library(QMA);
v <- volcano;
rownames(v) <- 1:nrow(v);
colnames(v) <- 1:ncol(v);
data <- getDataset(datasetCase = 2, processing = 0, fitnessMatrix = v);

# Histogram of deviations between the original
# data and processed rank-one approximation.
hist(data$m, 30);
```

```
# Example 2: Generate just the rank-one
# median estimates for the same matrix v.
estimates <- QMEstimate(fitness = v, p = c(0.5, 0.5));
```
